# Celiac Disease in Children With Type 1 Diabetes: The Usefulness of Screening— 25 years of Experience in a Single Center

**DOI:** 10.1155/pedi/4717290

**Published:** 2025-08-01

**Authors:** Roland Schweizer, Julia I. Bung, David Majer, Franziska Liebrich, Susann Herrlich, Andreas Neu, Julian Ziegler

**Affiliations:** Pediatric Diabetology, University Children's Hospital, Tübingen, Germany

## Abstract

**Objective:** Children with type 1 diabetes (T1D) have an increased risk of developing additional autoimmune diseases. The risk of developing celiac disease (CD) is 3–4 times higher in children with T1D. Guidelines recommend regular screening for transglutaminase antibodies (TgAbs) in T1D children. CD could be an additional burden for T1D children as both diseases affect food intake. We describe the screening practice for CD during the last 25 years in our outpatient clinic in children with T1D.

**Methods:** We retrospectively analyzed the development of CD-specific antibodies in our children with T1D (diabetes onset since 1998). We did not routinely recommend endoscopy when CD-specific antibodies (TgAb, endomysium [EAb], and gliadin) were positive and patients had no CD-specific symptoms.

**Results:** We analyzed 304 patients. In total 122 had CD-specific antibodies. In 98 of them, they disappeared after a short time or had been only slightly elevated. The diagnosis of CD was confirmed in 12. All 12 showed CD-specific symptoms, such as failure to thrive, anemia, hypoglycemia, or gastrointestinal problems. In six patients, even severely elevated EAb and/or TgAb disappeared on average after 7.1 years (range 4.9–13.5 years) on gluten-containing diet. The remaining six had antibodies without CD-specific symptoms by the end of the observation period. In this group the duration of antibody-positivity was 4 years (range 1.8–11.6 years).

**Conclusion:** We conclude that even highly elevated CD-specific antibodies can disappear in children with T1D and that screening for CD-specific antibodies is therefore only useful in symptomatic children with T1D.

## 1. Introduction

Children with type 1 diabetes (T1D) have an increased risk of developing additional autoimmune diseases, such as celiac disease (CD) or autoimmune endocrinopathies, for example, Hashimoto's thyroiditis. The risk of developing CD is 3–4 times higher in children with T1D compared to other children [1, 2]. Since the late 1990s, there has been an ongoing controversy about whether or not screening for CD in children with T1D is appropriate. CD and T1D both affect food intake and therefore increase the burden of disease. If left untreated, CD can also affect blood glucose control in diabetes, as patients may experience more unstable blood glucose levels and more frequent hypoglycemia [3]. On the other hand, a necessary gluten-free diet can also be unfavorable for blood glucose levels, as it is associated with faster-acting carbohydrates and can therefore also worsen metabolic control [4, 5]. Additionally, it has also been described in the literature that adults with CD who do not adhere to their diet appear to have an increased risk of intestinal lymphoma [3]. The guidelines therefore recommend regular screening for CD (e.g., every 2 years) [6–9], but so far there have been no studies to determine whether this is necessary. In other words, there is no evidence that screening for CD-specific antibodies in T1D prevents these children from harm. We therefore attempt to describe the experience of the last 25 years in our cohort of children and adolescents with T1D, in which we have carried out regular 2-yearly screening for CD by determining celiac-specific antibodies. Contrary to the recommendations in guidelines, we did not regularly initiate further diagnostics (e.g., endoscopy) for our children if CD-specific antibodies were detected and the child showed no CD-specific symptoms together with good diabetes control.

## 2. Patients and Methods

The study was approved by the Ethics committee of the Medical Faculty of the University of Tübingen (Germany). The Approval Number is 815/2021BO2.

We retrospectively analyzed 304 children and adolescents with T1D. The patients had their diabetes onset in the years from 1998 to 2022. The available data of the children up to the end of 2023 were included in the analysis. A blood sample from the patients was tested for CD-specific antibodies at the onset of T1D and at 2-year intervals thereafter. We measured transglutaminase antibodies (TgAbs), endomysium antibodies (EAbs), and gliadin antibodies as well as immunoglobulin A. Since this was a retrospective analysis, not all three antibodies were measured at all time points in all patients, and laboratory methods changed during the study period. Using conversion factors, we attempted to adjust the titers and levels according to the cut-off values provided by the manufacturer, using the rule of three. If a date was not exactly known, we used the middle of the most probable month in which the event took place. If the antibodies were positive, it was checked whether the patient had symptoms of CD, including unstable blood glucose levels, failure to thrive, anemia, gastrointestinal symptoms, skin problems, and elevated liver enzymes. If no symptoms were present, the results were discussed with the patient and parents, and a decision was made as to whether or not a duodenal biopsy should be performed. In asymptomatic patients, no biopsy was performed. Further diagnostics were recommended for all symptomatic patients with positive CD-specific antibodies. First, the antibodies were determined again and then either a duodenal biopsy or, in the case of very high transglutaminase IgA antibodies (>10-fold upper limit), the determination of EAbs or a further TgAb determination in a second independent blood sample. The diagnosis was then made in accordance with the internationally and nationally valid guidelines according to the MARSH classification or solely on the basis of elevated antibodies [10].

Based on the results of the diagnostics performed, the patients were divided into the following groups:

  Group 1: Patients who had elevated CD-specific antibodies at any time (EAbs 1-fold positive or titer <1:200, or TgAbs <40 U/L (reference <15 U/L), or positive gliadin antibodies).
  Group 2: Patients with elevated CD-specific antibodies in whom a biopsy would have been indicated according to the guidelines [10], but who did not show any CD-specific symptoms (repeated EAbs ≥2-fold positive or titers ≥1:200 and TgAbs repeatedly >40 U/L). The antibodies persisted until the end of the study period or until transfer to adult care.
  Group 3: Patients with elevated antibodies (like group 2), without symptoms, in whom the antibodies disappeared on a gluten-containing diet.
  Group 4: Patients with CD, symptoms of CD, and thereafter a gluten-free diet (repeated EAbs ≥2-fold positive or titer ≥1:200 and TgAbs repeatedly >40 U/L, positive biopsy or repeated extremely elevated antibodies).

According to the guidelines, a duodenal biopsy may be indicated for the diagnosis of CD if the transglutaminase and/or endomysium IgA antibodies are positive in two independent blood samples. If the transglutaminase IgA antibodies are higher than 10 times the upper normal limit, the diagnosis of CD, and thus the indication for a gluten-free diet can be made without a duodenal biopsy. In patients with transglutaminase IgA antibodies <10 times the upper limit of normal, the diagnosis of CD is established by a duodenal biopsy in which the abnormalities in the biopsy reach MARSH stage ≥2 in four samples from the duodenal mucosa [10].

The patient data were taken from the patient file (paper or digital). The blood results were taken from the laboratory program or, in the case of older patients, from digitalized archived laboratory records in the electronic patient file. The following data were entered into a database: date of diabetes onset, date of transfer to adult care (last visit to pediatric diabetology), height, weight, blood tests, HbA1c, type of therapy (CSII and ICT), insulin dose per kg body weight, diabetes complications, other comorbidities and CD-specific antibodies, date of first appearance of CD-specific antibodies, and date of first appearance of CD-specific symptoms, if applicable. The median and range were used to describe the group characteristics. To test for significant differences, the *t*-test was used if the values were normally distributed. Otherwise, the Mann–Whitney *U*-test or Wilcoxon test were used.

## 3. Results

**Figure 1:**
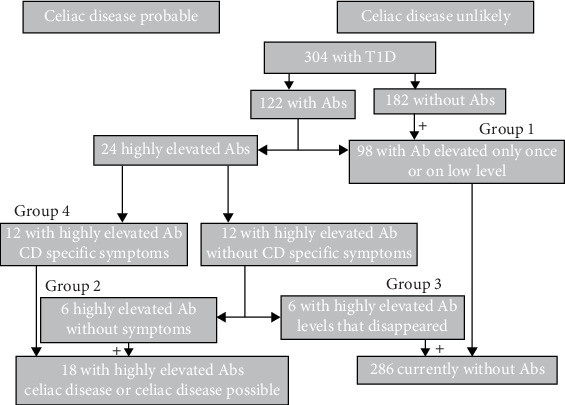
Breakdown of patients according to celiac disease specific antibody status. Ab, antibody; Abs, antibodies; T1D, type 1 diabetes.

We analyzed 304 patients with T1D. A total of 122 children and adolescents developed positive or elevated (above the threshold) CD-specific antibodies (all, endomysium, transglutaminase, and/or gliadin antibodies) during the observation period. In 98 of them, the antibodies were elevated only once or only marginal, sometimes for several times (group 1). The 98 children with once or low-level antibodies were distributed as follows: two children had higher antibodies once, six had low level antibodies once, 90 had low level antibodies for several times. Increased antibodies in this group were in 96 patients low level EAbs (<1:200) and in the two children with higher antibodies once the transglutaminase IgA-antibodies in one child and transglutaminase-IgG-antibodies in the other child. These children did not show symptoms of CD. The remaining 24 had repeatedly elevated CD-specific antibodies. The diagnosis of CD was made in 12 of these children (group 4). Ten of them were diagnosed by biopsy, two had antibody constellations that allowed a diagnosis based on the antibody titer alone. In six of the remaining 12, the elevated antibodies disappeared without a gluten-free diet (group 3). The other six patients still had elevated antibodies at the end of the observation period, but showed no CD-specific symptoms during the entire observation period (group 2).  Figure 1 shows the categorization into the four groups. The characteristics of the three groups with repeatedly elevated antibodies are shown in detail in Table 1. The patients who were diagnosed with CD had celiac-specific antibodies at a younger age and had the diabetes onset at a significantly younger age. Height, weight, and BMI did not differ significantly between the three groups at the time when antibodies were positive for the first time. All 12 children with CD showed symptoms associated with CD. Gastrointestinal symptoms occurred most frequently (eight patients). In four patients, the accompanying symptom was an unstable blood glucose level with a tendency to hypoglycemia, two of these had severe hypoglycemia, two showed failure to thrive, and two had microcytic anemia in the blood count. Four patients had two of the symptoms described. One patient was first diagnosed in adult care, but had symptoms and antibodies during pediatric care. In the children in whom the antibodies disappeared again (group 3), the antibodies were detectable for a median of 7.1 years (range 4.9–13.5 years). In patients who still had elevated antibodies at the end of the study period (group 2), these were detectable for a median of 4.0 years (range 1.8–11.6 years). The median time between the first appearance of antibodies and the appearance of symptoms associated with CD was 3.1 years (range 0–13.1 years) in the group with CD (group 4). The levels of transglutaminase and endomysium antibodies were highest on average in children with CD (group 4) and showed a trend toward lower levels in children with antibodies without CD-specific symptoms (group 2). They were still somewhat lower in those where the antibodies had disappeared (group 3). However, there was significant overlap in the height of the antibody titers in individual patients. The differences were not significant due to the small number of patients (Table 1). The highest values for individual patients in all three groups were similar. Eleven (46%) of the patients had positive antibodies at the onset of diabetes. Five (42%) in the group with CD, four (67%) in the group in which the antibodies disappeared, and two (33%) in the group with still persistent antibodies, without CD-specific symptoms.

## 4. Discussion

To our knowledge, this study is the first to show that CD-specific antibodies can disappear in children with T1D, even when they are highly elevated. Castellaneta et al. [11] described that TgAbs can disappear in children when detected at low levels, but not at high levels. This finding that antibodies disappear when levels are not high is consistent with the 98 patients in our cohort (group 1) who had only slightly elevated levels or briefly elevated levels. However, in our cohort, the overall proportion of patients with antibodies was higher. Castellaneta et al. [11] reported 65 out of 446 patients with elevated levels, whereas in our cohort it was 122 out of 304 children and adolescents with T1D. The difference could be due to the fact that we screened for all antibodies (transglutaminase, endomysium, and gliadin antibodies) and Castellaneta only for TgAbs. Waisbourd-Zinman et al. [12] published similar results. They found that spontaneous normalization of antiTgAb levels is common in children with T1D mellitus. They concluded, “physicians treating children with T1D and mildly elevated antiTgAb levels might consider a 12-month serologic follow-up on a gluten-containing diet instead of an immediate duodenal biopsy.” The difference to our results is that we found high-titer positive antibodies against transglutaminase and endomysium in our children and adolescents, which normalized under a gluten-containing diet, and that the antibodies in our patients were often positive for several years. On the other side, we cannot rule out the unlikely possibility that children with CD-specific symptoms without noticeable antibodies with normal IgA have not been diagnosed.

Some authors are concerned that people with T1D may have subclinical CD [13]. However, the symptoms described by the authors as subclinical CD in children with T1D are those that we have recognized as symptoms of CD, such as failure to thrive or hypoglycemia. Kamrath et al. [14] described in a population-based study with data from the DPV registry (prospective diabetes follow-up registry initiative) that in children with positive celiac serology at the time of diabetes onset, histologic confirmation of CD after 6–36 months had no adverse effects on mean HbA1c, the occurrence of diabetes complications and the achievement of seronegativity for antitransglutaminase titers compared to early biopsy within the first 6 months. This raises the question of whether screening for CD in asymptomatic children and adolescents with T1D is appropriate. Currently, such a screening is recommended in national and international guidelines [6, 7] at the onset of diabetes and thereafter at annual or biennial intervals. There is also no screening for CD-specific antibodies in the asymptomatic general population. With a CD prevalence of about 1% in the general population and a CD prevalence in children and adolescents with diabetes of 4.7% [1] (3.9% in our study), the attributive risk of CD in children and adolescents with T1D is 3.7% (2.9% in our study). Thus, 27 (34 in our study) children and adolescents with T1D would need to be screened to detect one child with symptomatic CD. If the celiac-specific antibodies in our cohort had only been determined in children with symptoms suggestive of CD, all children with CD would have been identified. The development of symptoms sometimes occurred later than the first appearance of antibodies. It is therefore possible that some of our children in group 2 may still develop CD-specific symptoms after the study period, especially those who were younger and for whom the time with positive antibodies was shorter. On the other hand, it is also possible that the antibodies may disappear in some children in group 2.

One of the main arguments for screening for CD is the fact that the risk of gastrointestinal lymphoma is increased in adults with CD. This has been demonstrated in long-term untreated CD patients and also in patients with refractory CD [15]. Nevertheless, Lang-Muritano et al. [3] questioned routine screening for CD in children with T1D as early as 2002, as the risk of lymphoma is low. They found no patients with T1D in a cohort of adult patients with gastrointestinal lymphomas [16]. Catassi et al. [17] demonstrated a threefold increased risk of gastrointestinal lymphoma in CD and concluded that this association does not represent a sufficient risk to justify early mass screening for CD in the general population. If CD is diagnosed in T1D, the following factors must be considered. It could be more difficult for children with T1D to control their blood glucose levels on a gluten-free diet [4, 5]. A gluten-free diet can also be a financial burden, as a gluten-free diet is more expensive and there is less choice of food. This can lead to a decrease in quality of life in the patients and the family. As a result, a diagnosis of CD can cause families additional psychological stress and they have to bear the burden of a second chronic illness. These facts are particularly important if the diagnosis of CD is made without reasonable justification.

The weakness of our study is the low number of children with CD and with persistently elevated CD-specific antibodies. The patient data were collected retrospectively, so that some information is not available for all patients, for example, the exact start of gluten-free diet. The strength of the study is the long observation period, during which the patients were advised in the same way, meaning that a biopsy was not always recommended immediately if antibodies were elevated. This made it possible to observe antibody courses in which even high CD-specific antibodies disappeared again.

## 5. Conclusion

Taking these arguments and evidence together, there is no reason to screen asymptomatic children and adolescents with T1D for CD. It should therefore be discussed whether it is necessary to continue to recommend screening in national and international guidelines. Instead, it should be emphasized that children with typical symptoms (susceptibility to hypoglycemia, failure to thrive, anemia, gastrointestinal problems, etc.) should be tested for CD by determining antibodies. This study may lead to a debate on screening for CD and may serve as a starting point for a paradigm shift in recommendations concerning this matter.

## Figures and Tables

**Table 1:** Characteristics of patients with celiac disease specific antibodies.

	Patients with celiac disease (group 4), *N* = 12			Patients with persistant Abs (group 2), *N* = 6			Patients with Abs which disappeared (group 3), *N* = 6			Group 4 vs. 2 + 3
	Median	Min.	Max.	Median	Min.	Max.	Median	Min.	Max.	*p*
Age (years) at diabetes onset	3.4	0.9	11.1	6.1	1.9	14.7	7.2	3.1	12.1	<0.05
Age (years) at first time postive Abs	4.8	1.5	17.5	9.2	5.1	16.3	8.8	4.8	12.1	n.s.
HbA1c (%) at first time positve Abs	7.7	6.7	13.4	9.6	7.2	14.0	7.7	7.1	13.1	n.s.
Height (SDS) at first time positve Abs	−0.19	−1.90	2.85	0.53	−0.69	0.98	−0.19	−0.46	1.16	n.s.
Weight (SDS) at first time Abs	−0.05	−1.69	3.02	0.24	−0.48	1.17	0.72	−1.07	2.10	n.s.
BMI (SDS) at first time Abs	−0.28	−0.95	1.03	0.16	−1.94	0.57	0.48	−1.20	1.02	n.s.
Time (years) until disappearence of Abs/end of study period	—	—	—	4.0	1.8	11.6	7.1	4.9	13.5	—
Diabetes duration (years) at first appearence of Abs	0.6	0.0	12.7	2.9	0.0	5.5	0.0	0.0	3.2	n.s.
Highest EM-IgA-Ab levels	1600	100	6400	3200	200	3200	600	100	3200	n.s.
Highest TglgA-Ab levels	117.4	0.0	3724.1	130.7	50.0	195.8	49.2	7.1	129.3	n.s.

*Note: p*, level of significance.

Abbreviations: Ab, antibody; Abs, antibodies; EM-IgA-Abs, endomysium-immunoglobulin-A antibodies; n.s., not significant; TgIgA-Abs, transglutaminase-immunoglobulin-A antibodies.

## Data Availability

The data that support the findings of this study are available upon request from the corresponding author. The data are not publicly available due to privacy or ethical restrictions.
